# Overcoming chromatin barriers

**DOI:** 10.7554/eLife.50761

**Published:** 2019-09-13

**Authors:** Babette E de Jong, John van Noort

**Affiliations:** Biological and Soft Matter Physics, Huygens–Kamerlingh Onnes LaboratoryLeiden UniversityLeidenNetherlands

**Keywords:** nucleosome transcription, high-resolution optical tweezers, histone variant H2A.Z, epigenetic modifications, histone ubiquitination, transcription regulation, Human, *Xenopus*, *E. coli*

## Abstract

Single-molecule experiments reveal the dynamics of transcription through a nucleosome with single-base-pair accuracy.

**Related research article** Chen Z, Gabizon R, Brown AI, Lee A, Song A, Diaz-Celis C, Kaplan CD, Koslover EF, Yao T, Bustamante C. 2019. High-resolution and high-accuracy topographic and transcriptional maps of the nucleosome barrier. *eLife* **8**:e48281. doi: 10.7554/eLife.48281

A human cell contains about two meters of DNA, which is organized into a macromolecular complex called chromatin. The basic unit of chromatin is the nucleosome – a structure that consists of 147 base pairs wrapped around eight histone proteins – and three-quarters of our DNA is found in nucleosomes (Luger et al., 1997). The formation of chromatin compacts the DNA by a factor of about 10,000, allowing it to fit in the cell nucleus. However, DNA also needs to be readily accessible to various enzymes to allow genes to be transcribed into messenger RNA (mRNA) molecules, which can then be translated to produce proteins.

The 'unpacking' of DNA that occurs during transcription involves it being unwrapped from the nucleosome and then separated into two strands (a process called DNA melting). mRNA molecules are then produced by an enzyme called RNA polymerase II (Pol II) as it moves along the DNA. A nucleosome presents an obstacle for Pol II and the production of mRNA, but the unwrapping of short lengths of DNA allows Pol II to continue moving and producing mRNA (Ehara et al., 2019; Kujirai et al., 2018).

To fully understand the changes in the complex formed by Pol II and a nucleosome during transcription we must be able to relate these changes to a detailed map of the energy landscape for the transcription process. Now, in eLife, Carlos Bustamante, Tingting Yao and co-workers – including Zhijie Chen and Ronen Gabizon, both from the University of California Berkeley, as joint first authors – report that they have produced a map of the energy landscape based on measurements made with millisecond time resolution and sub-nanometer accuracy (Chen et al., 2019).

The experiments involved single-molecule assays in which 'optical tweezers' were used to apply forces to strands of DNA. In the first assay, a DNA unzipping assay, the two strands of a DNA molecules are slowly pulled apart (Figure 1A). It takes slightly more energy to melt a GC base pair than an AT base pair, so the resulting 'rupture profile' (which is a plot of force versus position; Figure 1B) depends on the DNA sequence. Moreover, a decade ago Michelle Wang and co-workers at Cornell University showed that the energy needed to unzip the DNA is higher when it is wrapped around a nucleosome. By careful analysis of the rupture profile, they were able to determine the energy landscape for nucleosome unwrapping (Hall et al., 2009).

**Figure 1.**
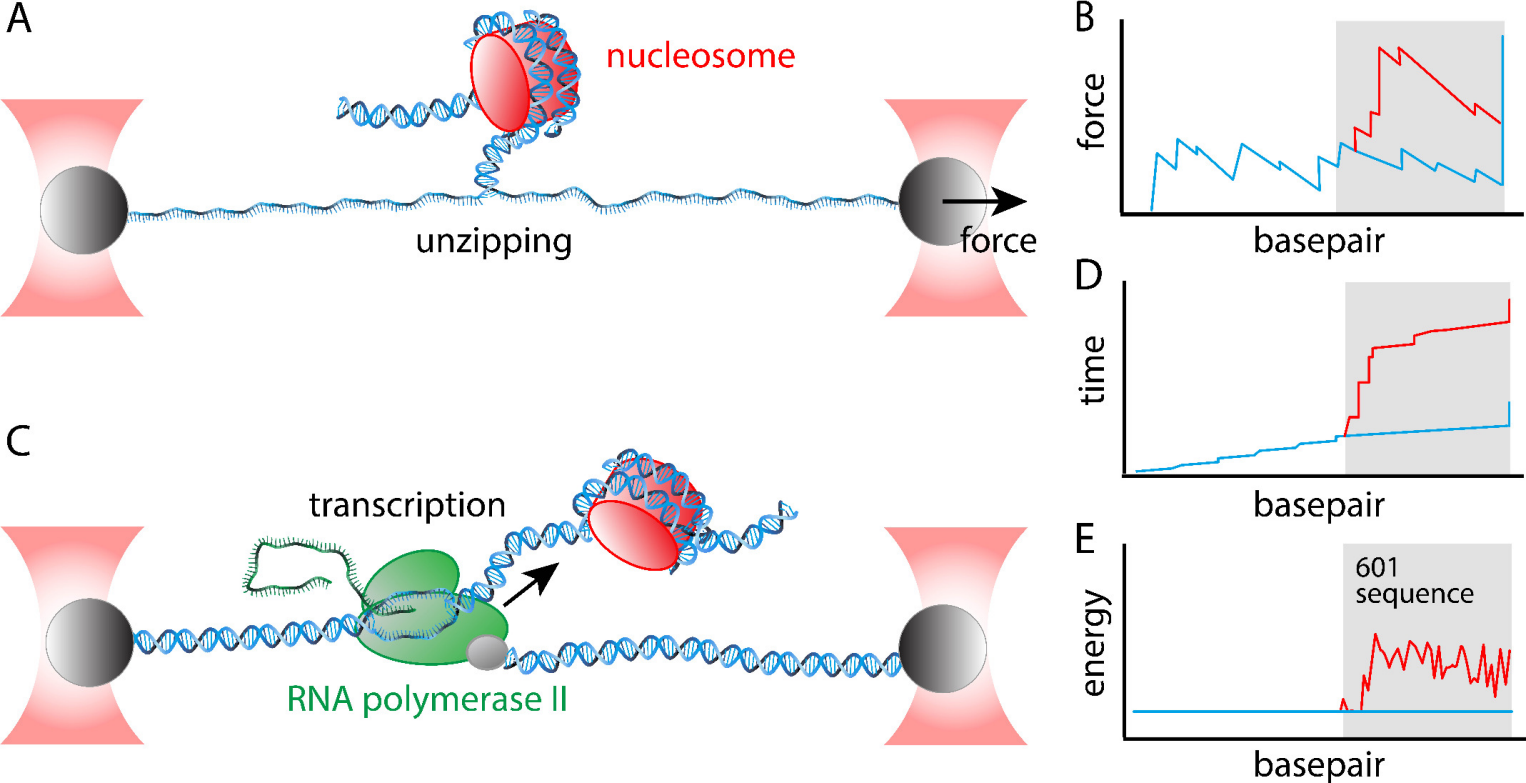
Determining the energy landscape for transcription through a nucleosome. Chen et al. combined two single-molecule assays – both of which relied on 'optical tweezers' – to the determine the energy landscape for transcription. Optical tweezer experiments involve attaching a polystyrene bead (grey) to a molecule of interest, and then using a focused laser beam (red) to move the bead and thus apply a force to the molecule. (**A**) In the unzipping assay optical tweezers are used to pull the two strands in a DNA molecule apart. (**B**) A plot of force versus base pair (which is essentially distance along the DNA molecule) is jagged because it takes more energy to melt GC base pairs than AT base pairs (blue line). Nucleosomes also obstruct unzipping and increase the energy needed to melt the base pairs (red line). The grey area indicates the location of the Widom 601 positioning sequence. (**C**) In a transcription assay, one of the optical tweezers holds the 5' end of the DNA molecule that is being transcribed by a Pol II enzyme (green); the other optical tweezer applies a force to the enzyme itself. The mRNA molecule is the green structure to the left of the Pol II enzyme. (**D**) Plotting time versus base pair when a constant force is applied by the optical tweezers shows that there are small, but regular pauses in transcription (indicated by the short vertical regions in the blue line) as a consequence of the stalling sequences that were introduced in the DNA; there are no obvious pauses during transcription of the Widom 601 sequence (blue line, grey background), but there are several lengthy pauses during transcription through the nucleosome (red line, grey background). (**E**) By combining the rupture profile (B) and the transcription time trace (D) in a thermodynamic model that includes the geometry of the nucleosome-Pol II complex, Chen et al. were able to extract the energy landscape, which is smooth in the absence of the nucleosome (blue line), and which contains multiple peaks and valleys in the presence of the nucleosome (red line).

Around the same time, Bustamante and co-workers developed a single-molecule transcription assay in which one tweezer holds the DNA molecule being transcribed, and a second tweezer applied a force to the Pol II enzyme (Figure 1C). This assay, which allows the progress of the transcription reaction to be followed in real time (Figure 1D), showed that the velocity of transcription depended on the DNA sequence, and that certain sequences could cause Pol II to stall, or even to start moving backwards (Hodges et al., 2009; Figure 1D). More recently this assay was used to show that the presence of a nucleosome caused Pol II to slow down (Dangkulwanich et al., 2013).

In the latest work, the accuracy of both assays has been improved and their outputs have been combined to produce a highly-detailed map of the energy landscape for transcription through a nucleosome (Figure 1E). The process of transcription can be thought of as a Brownian ratchet: in this model the Pol II enzyme 'hops' along an energy landscape in the forward direction when it has enough thermal energy to overcome the next peak in the landscape, and is far less likely to move backwards. The more peaks there are in the landscape, and the higher these peaks are, the longer the Pol II enzyme dwells in the valleys between peaks. A major challenge is to distinguish between three effects that depend on the DNA sequence: DNA unwrapping from the nucleosome; DNA melting (ie, the fact that it takes more energy to melt a GC base pair); and alternative transcription pathways.

To be able to combine data from the two assays, Chen et al. introduced a 'molecular ruler' into the DNA substrate ahead of the specific sequence of bases (called a Widom 601 sequence; Lowary and Widom, 1998) that forces the nucleosome into a defined position. In the unzipping assay the ruler consisted of a second copy of the 601 sequence; in the transcription assay it was a regular repeat of a sequence that is known to stall Pol II. This improved the accuracy with which they could trace transcription through the nucleosome by a factor of 10.

Chen et al. started by performing an unzipping assay on a sample of DNA that contained two positioning sequences, and then on a sample that contained a positioning sequence and a nucleosome. Next, they performed a transcription assay on a sample that contained the array of stalling sequences and a nucleosome. They found that the first 20 base pairs of nucleosomal DNA were easily transcribed, but that Pol II experienced substantial delays in the remaining part of the nucleosome. This is reflected in the energy landscape they determined from the data (Figure 1E).

As mentioned above, a nucleosome contains eight histones. Most nucleosomes contain pairs of regular H2A, H2B, H3 and H4 histones. However, some nucleosomes contain a variant of H2A called H2A.Z and/or a ubiquitinated version of H2B called uH2B, and these epigenetic changes are known to influence transcription. Chen et al. observed increased dwell times for transcription through nucleosomes with H2A.Z or uH2B, compared with the dwell times for transcription through canonical nucleosomes, which suggests that these variants might directly regulate transcription in vivo. However, establishing a direct link is not trivial because it has been shown that H2A.Z incorporation lowers transcription barriers in vivo (Weber et al., 2014). Moreover, there is evidence to suggest that the ubiquitination of H2B enhances rather than impedes transcription, possibly by recruiting elongation factors. Thus, while resolving the energy landscape sheds light on the mechanisms behind transcription regulation, it does not reveal the whole story.

Transcription regulation by nucleosomes is also highly context-specific: for example, in vivo experiments suggest that the barrier presented by a nucleosome depends on the exact location of that nucleosome within the genome (Teves et al., 2014). This might be due to the presence of modified nucleosomes (such as H2A.Z and uH2B), but other factors are equally likely to play a role. The DNA sequence itself also affects nucleosome stability, and it will be interesting to see if the energy landscape changes when sequences other than the artificial Widom 601 sequence are used. It might not be a coincidence that the highest barrier for transcription within the nucleosome coincides with the location of the strongest nucleosome positioning signal in this sequence (Segal et al., 2006). Other context-specific factors that may modulate – and potentially dominate – the transcription landscape include proteins that 'remodel' chromatin, transcription factors, DNA supercoils and the formation of higher-order chromatin structures.

The work of Chen et al. highlights the unique contributions that single-molecule studies can make to structural biology. And by building on this work, more insights into the regulation of transcription by nucleosomes are within reach.
